# *BrCPS1* Function in Leafy Head Formation Was Verified by Two Allelic Mutations in Chinese Cabbage (*Brassica rapa* L. ssp. *pekinensis*)

**DOI:** 10.3389/fpls.2022.889798

**Published:** 2022-07-12

**Authors:** Yue Gao, Gaoyang Qu, Shengnan Huang, Zhiyong Liu, Wei Fu, Meidi Zhang, Hui Feng

**Affiliations:** ^1^College of Bioscience and Biotechnology, Shenyang Agricultural University, Shenyang, China; ^2^Liaoning Key Laboratory of Genetics and Breeding for Cruciferous Vegetable Crops, College of Horticulture, Shenyang Agricultural University, Shenyang, China

**Keywords:** Chinese cabbage, leafy head formation, allelic mutations, CPS1, MutMap

## Abstract

The formation of the leafy heads of Chinese cabbage is an important agricultural factor because it directly affects yield. In this study, we identified two allelic non-heading mutants, *nhm4-1* and *nhm4-2*, from an ethyl methanesulfonate mutagenic population of a heading Chinese cabbage double haploid line “FT.” Using MutMap, Kompetitive Allele-Specific PCR genotyping, and map-based cloning, we found that *BraA09g001440.3C* was the causal gene for the mutants. *BraA09g001440.3C* encodes an *ent*-copalyl diphosphate synthase 1 involved in gibberellin biosynthesis. A single non-synonymous SNP in the seventh and fourth exons of *BraA09g001440.3C* was responsible for the *nhm4-1* and *nhm4-2* mutant phenotypes, respectively. Compared with the wild-type “FT,” the gibberellin content in the mutant leaves was significantly reduced. Both mutants showed a tendency to form leafy heads after exogenous GA_3_ treatment. The two non-heading mutants and the work presented herein demonstrate that gibberellin is related to leafy head formation in Chinese cabbage.

## Introduction

The leafy head is a unique organ in Chinese cabbage, and the shape, size, uniformity, and density of the leafy head directly affect its commercial value (Zhang et al., 2021). The formation of Chinese cabbage leafy heads is biologically complex, including four developmental stages, the seedling, rosette, folding, and heading stages. Leaves change from flat to upward facing in the rosette stage and start to fold in the folding stage, eventually forming a leafy head in the heading stage (He et al., 2000). The formation of leafy heads is affected by various factors, including the temperature, light intensity, auxin concentration, carbon to nitrogen ratio, and leafy shape (Ito and Kato, 1957; He et al., 2000; Mao et al., 2014).

The mechanism of Chinese cabbage leafy head formation is complex, and there is no current hypothesis that can fully explain the mechanism. However, in recent years, some genes related to leafy head formation in Chinese cabbage have been reported. The target gene *BrpSPL9-2* of microRNA brp-miR156 regulates the time of Chinese cabbage heading by shortening the seedling and rosette stages (Wang et al., 2014). *BrBRX* genes (*BrBRX.1*, *BrBRX.2*, and *BrBRX.3*) control leaf morphological development, and *BrBRX.1* and *BrBRX.2* genes had similar expression patterns and may be involved in the formation of leafy heads in *Brassica rapa* (Cheng et al., 2016; Zhang et al., 2021). Yu et al. (2019) explored the expression of *BrAN3* before and after the formation of Chinese cabbage leafy heads and found that *BrAN3* was significantly expressed in the rosette and heading leaves. According to the different expression patterns of different leaf locations, it was confirmed that *BrAN3* could induce the formation of leafy head. Ren et al. (2020) discovered that *BcpLH* regulates the timing of leafy head formation by integrating important miRNAs.

Plant hormones play important roles in the formation of leafy heads. Cheng et al. (2016) found that four plant hormones (cytokinin, auxin, gibberellin, and jasmonic acid) regulate the formation and development of the leafy head in Chinese cabbage. Yu et al. (2019) reported similar results and proposed that *BrAN3* can induce GA, BR, and SA signaling pathways, thereby inhibiting the formation of Chinese cabbage leafy heads. Gao et al. (2017) revealed that auxin transport genes (*BrAUX*/*LAX*, *BrPIN*, and *BrPGP*) play an important role in leafy head formation in Chinese cabbage by genome-wide annotation and bioinformatics analysis. Li et al. (2019) found that both auxin and abscisic acid signaling pathways play important roles in regulating early leafy head formation. In our previous study (Gao et al., 2020), we found that the non-heading phenotype of Chinese cabbage is caused by mutations in the *BraA07g042410.3C* gene, which encodes *ent*-kaurene synthase (*KS*), a key enzyme involved in gibberellin (GA) biosynthesis. This result indicates that the GA content in Chinese cabbage leaves is related to the formation of leafy heads. The above research results provide a basis for studying the mechanisms of the hormones involved in Chinese cabbage leafy head formation, and further research on genes related to leafy head formation can improve our understanding of the molecular mechanism of leafy head development.

In this study, we found two non-heading mutants (*nhm4-1* and *nhm4-2*) whose phenotypes were consistent with each other. Allelism testing proved that the mutant genes of *nhm4-1* and *nhm4-2* were allelic. MutMap, Kompetitive Allele-Specific PCR (KASP), and map-based cloning analyses were performed to identify the candidate gene of the mutants. We demonstrated that *BraA09g001440.3C* (*BrCPS1*), which encodes an *ent*-copalyl diphosphate synthase 1 (*CPS1*) involved in GA biosynthesis, was the candidate gene. The function of *BrCPS1* in the leafy head formation of Chinese cabbage was further confirmed by the determination of the GA content in the leaves of two allelic mutants and the spraying with exogenous GA_3_. These results provide information for understanding the formation mechanism of leafy heads in Chinese cabbage.

## Materials and Methods

### Plant Materials

In our previous study (Gao et al., 2020), 14 non-heading mutant plants were harvested in an EMS-induced mutagenic population. For this study, we selected two mutants with extremely similar phenotypes, *nhm4-1* and *nhm4-2*.

### Genetic Analysis

For genetic analysis, the mutants were crossed with the wild-type “FT” to obtain F_1_, F_2_, and BC_1_ populations. To investigate the genetic characteristics of the mutants, we recorded the phenotypes of each plant in each generation and analyzed the separation rate of the F_2_ and BC_1_ populations using the Chi-square (*χ*^2^) test.

### Allelism Test Between *nhm4-1* and *nhm4-2*

To detect the allelism of the two mutant genes, we conducted an allelism test. Mutants *nhm4-1* and *nhm4-2* were used as parents for hybridization, and the phenotypes of their hybrid progeny were observed and recorded.

### Mutmap Analysis to Determine the Candidate Gene

A modified MutMap method (Abe et al., 2012) was applied for fine mapping and identification of candidate genes for *nhm4-1*. For MutMap, the DNA of 50 mutant plants in the F_2_ population was mixed equally as the mutant pool. DNA from the two parental plants and the mutant pool asextracted from fresh leaves at the rosette stage using a DNA secure plant kit (Tiangen, Beijing, China) and resequenced with a NovaSeq 6,000 sequencer (Illumina, San Diego, CA, United States of America).

Low-quality data were filtered from the raw data according to the filtering criteria of our previous study (Gao et al., 2020) to obtain clean reads. Clean reads were mapped to reference genome sequences using BWA software (Li and Durbin, 2010), and SAMtools (Li et al., 2009) was used to sort the alignment file. Insertions and deletions (INDELs) and single-nucleotide polymorphisms (SNPs) were identified using GATK software (McKenna et al., 2010) and ANNOVAR software (Wang et al., 2010). Circos software (Krzywinski et al., 2009) was used to map variation information in the genome. The ΔSNP index across the chromosomes of the *B. rapa* genome was obtained using sliding-window analysis (with a five-SNP window size and one SNP for each step).

### SNP Genotyping by KASP

Kompetitive Allele-Specific PCR was developed for the genotypic assay to detect the co-segregation of each SNP and to confirm the *nhm4-1* candidate gene. The allele-specific primers used are shown in Supplementary Table S1. A total of 184 F_2_ plants were used for KASP genotyping. Of these, 48 plants showed a mutant phenotype and 136 exhibited the wild-type phenotype. The assay was carried out at the Vegetable Research Center of the Agriculture and Forestry Academy in Beijing.

### Cloning and Sequence Analysis

The coding sequences of candidate genes were amplified with specific primers (Supplementary Table S2) in the wild-type “FT,” *nhm4-1* mutant, and *nhm4-2* mutant plants. Primer 5.0 was used to design specific primers and perform gene cloning following the methods of Gao et al. (2020). Sequencing was performed using the Sanger method at GENEWIZ (Suzhou, China). The sequences were aligned using DNAMAN V6 software (Lynnon BioSoft, Montreal, QC, Canada).

### Enzyme Activity Assays

*Ent*-Copalyl diphosphate synthase 1 activity in the leaves from the wild-type and *nhm4-1* mutant plants was evaluated using the Plant CPS enzyme-linked immunosorbent assay (ELISA) Kit (Meimian Biotech Co., Ltd., Jiangsu, China) via a double antibody sandwich method following the manufacturer’s instructions. Each material was performed for three biological repeats, and three times the technical repeats were performed in each biological repeat.

### RNA Isolation and Quantitative Real-Time PCR

For analysis of the relative expression levels of the candidate gene, total RNA was extracted from the cotyledons, first true leaves, third true leaves, sixth true leaves, rosette leaves, and heading leaves of wild-type “FT,” mutant *nhm4-1*, and mutant *nhm4-2* plants using an RNA extraction kit (Aidlab, Beijing, China). First-strand cDNA was synthesized using the FastQuant RT kit (Tiangen, Beijing, China) and quantitative real-time PCR (qRT-PCR) was performed using an Ultras SYBR Mixture (CWBIO, Beijing, China) and the Quant Studio 6 Flex Real-Time PCR System (ABI, Los Angeles, CA, United States of America). The *Actin* gene (F: 5′-ATCTACGAGGGTTATGCT-3′; R: 5′-CCACTGAGGACGATGTTT-3′) was used as the reference gene. Each experiment was independently performed with three technical replicates and three biological replicates. The relative gene expression levels were calculated using the 2^−ΔΔCt^ method (Livak and Schmittgen, 2001). The primer sequences used for qRT-PCR amplification are shown in Supplementary Table S3.

### Measurement of Endogenous GA Content and Treatment of Exogenous GA_3_

According to our previous methods (Gao et al., 2020), the content of endogenous GA in the leaves of wild-type “FT,” mutant *nhm4-1*, and mutant *nhm4-2* plants was determined by liquid chromatography-tandem mass chromatography (Chen et al., 2012). The response of the mutants *nhm4-1* and *nhm4-2* to GA was determined by spraying exogenous GA_3_ solution (500 mg/L). The plants were sprayed with GA_3_ at 3-day intervals once the cotyledons were fully expanded, and the treatments ended before the rosette stage. Seedlings sprayed with an equal volume of ddH_2_O without GA_3_ were used as controls.

## Results

### Phenotypic Characterization and Inheritance Analysis of *nhm4-1* and *nhm4-2*

**Figure 1:**
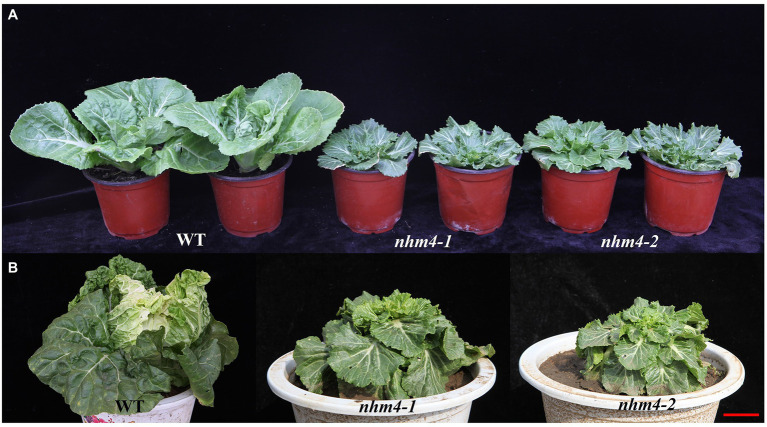
Phenotype observation of the wild-type “FT” and mutant plants. **(A)** Phenotypes of wild-type “FT,” mutant *nhm4-1*, and mutant *nhm4-2* plants during the rosette stage. **(B)** Phenotypes of wild-type “FT,” mutant *nhm4-1*, and mutant *nhm4-2* plants at the heading stage. Bar = 5 cm.

The phenotype of the mutant *nhm4-1* was highly consistent with that of the mutant *nhm4-2*. The leaves of the mutant plants showed geotropic growth throughout the developmental stages and could not form leafy heads at the heading stage, unlike those of the wild-type “FT” plants (Figure 1).

As shown in Table 1, the phenotype of all plants in the F_1_ generation was consistent with that of the wild-type “FT.” The segregation ratio of the F_2_ generation was 3:1, while that of the BC_1_ generation was approximately 1:1. These results indicate that the mutation phenotype of the *nhm4-1* and *nhm4-2* mutants was controlled by a single recessive nuclear gene.

**Table 1:** Genetic analysis of mutants *nhm4-1* and *nhm4-2.*

Generations	Total plants		Mutant plants		Wild type plants		Segregation ratio		*χ* 2	
	*nhm4-1*	*nhm4-2*	*nhm4-1*	*nhm4-2*	*nhm4-1*	*nhm4-2*	*nhm4-1*	*nhm4-2*	*nhm4-1*	*nhm4-2*
P_1_(WT)	50	50	0	0	50	50				
P_2_(*nhm4-1* or *nhm4-2*)	50	50	50	50	0	0				
F_1_(P_1_ × P_2_)	30	30	0	0	30	30				
F_1_(P_2_ × P_1_)	30	30	0	0	30	30				
BC_1_(F_1_ × P_1_)	60	70	0	0	60	70				
BC_1_(F_1_× P_2_)	85	70	45	32	40	38	1.12:1	1.18:1	0.18	0.35
F_2_	350	250	82	59	268	191	3.26:1	3.23:1	0.38	0.19

### Allelism Testing

**Figure 2:**
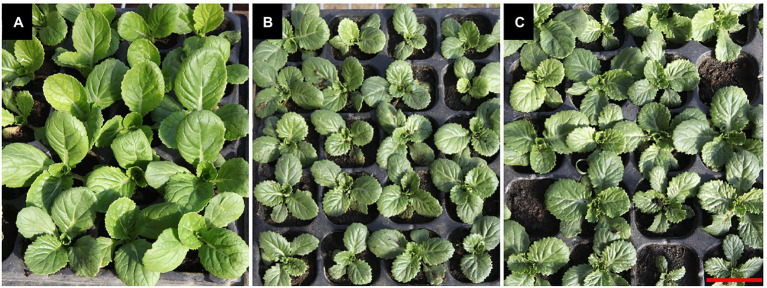
Allelism test of mutant *nhm4-1* and mutant *nhm4-2*. **(A)** Wild-type “FT.” **(B)** Mutant *nhm4-1* × *mutant nhm4-2*. **(C)** Mutant *nhm4-2* × mutant *nhm4-1*. The plants are shown at 35 days after sowing (DAS). Bar = 5 cm.

We crossed mutants *nhm4-1* and *nhm4-2* to detect the allelism of the two. Both F_1_ populations exhibited a mutant phenotype after hybridization, indicating that the mutant genes of *nhm4-1* and *nhm4-2* are allelic (Figure 2).

### Identification of the Candidate Gene Responsible for *nhm4-1*

The wild-type “FT,” the mutant *nhm4-1*, and a mutant pool containing 50 homozygous recessive F_2_ mutant plants were resequenced, resulting in 94,719,968, 118,123,996, and 270,862,892 clean reads, respectively. A total of 98.00, 98.43, and 98.91% of the clean reads in the wild-type “FT,” mutant *nhm4-1*, and mutant pools, respectively, were mapped to the Chinese cabbage v. 3.0 reference genome (BRAD^1^). Based on the alignment to the reference genome sequence, the mutation analysis software GATK (McKenna et al., 2010) was used to extract all potential polymorphic SNP sites in the genome. Circos software was used to draw the variation information on the genome, and it was found that SNP was mainly distributed on chromosome A03 and A09 (Supplementary Figure S1). This was followed by further filtering and screening, after which 1,587 high-quality SNPs were obtained.

**Figure 3:**
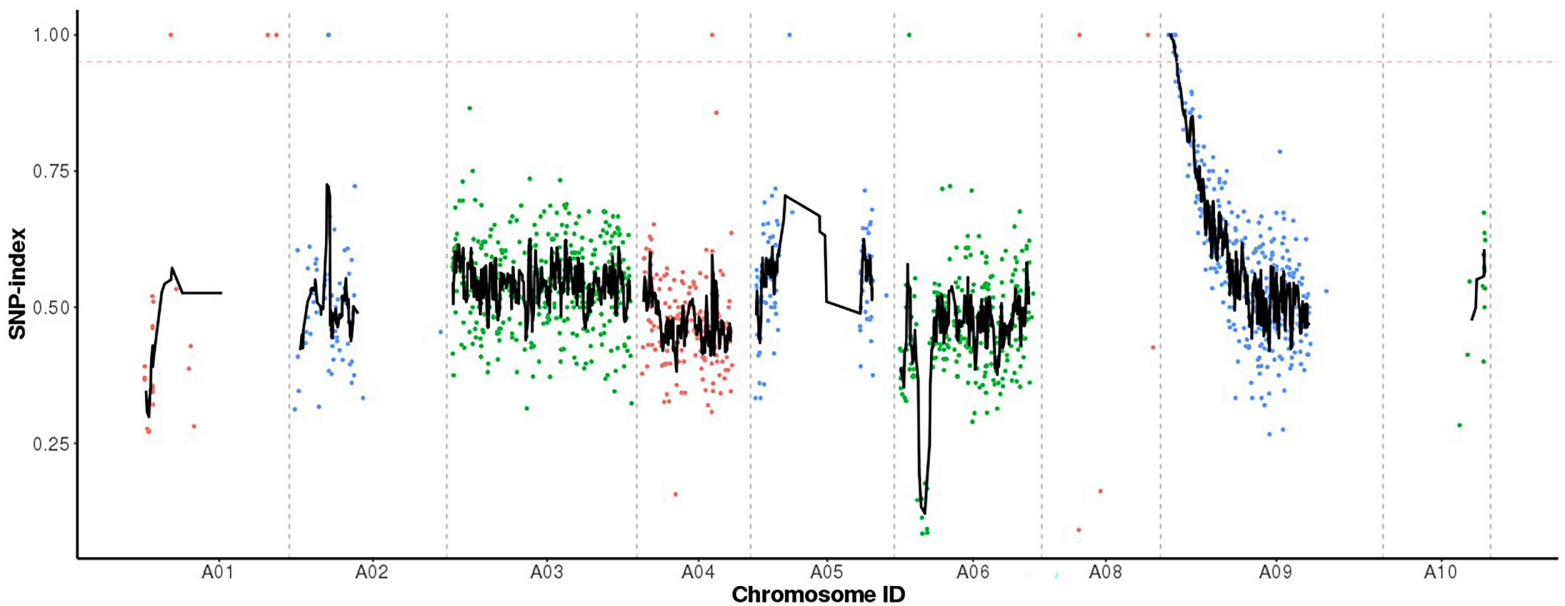
Identification of candidate gene by MutMap. The *X*-axis represents ten chromosomes, the *Y*-axis represents the SNP index value, and the dotted pink line is the index threshold (0.95).

When the SNP index was 0.95 as the threshold, we located a 1.59 Mb (867,020-2,457,084) candidate region on chromosome A09 (Figure 3). Five SNP mutations occurred in the exon, of which only two SNPs (SNP A09, 900,112 and SNP A09, 1,723,490) caused non-synonymous amino acid changes (Table 2). SNP A09, 900,112 (C–T) was located in the *BraA09g001440.3C* and SNP A09, 1,723,490 (G–A) was located in the *BraA09g002790.3C*.

**Table 2:** Candidate SNP information from the candidate region.

Chromosome	Position	Gene ID	Variation base	SNP index	Exon ID	Mutation type	Annotation information
A09	900,112	*BraA09g001440.3C*	C–T	1	Exon 7	Non-synonymous SNV	*ent*-copalyl diphosphate synthase
A09	1,639,305	*BraA09g002590.3C*	C–T	1	Exon 1	synonymous SNV	E3 ubiquitin-protein ligase RMA1
A09	1,723,490	*BraA09g002790.3C*	G–A	1	Exon 1	Non-synonymous SNV	Cell division cycle protein 123 homolog
A09	2,003,067	*BraA09g003180.3C*	G–A	0.98	Exon 1	synonymous SNV	Succinate dehydrogenase
A09	2,193,985	*BraA09g003550.3C*	G–A	0.96	Exon 9	synonymous SNV	–

### KASP Analysis

To confirm the candidate SNP, primers were designed based on the mutation information of these two SNPs and then applied to F_2_ populations. Genotyping analysis was performed using KASP, and the association between these two SNPs and the mutant phenotypes was verified. The genotypic assay showed that SNP A09, 900,112 of *BraA09g001440.3C* was the T:T genotype in the 48 mutant phenotypic plants and the C:T or C:C genotypes in the 136 wild-type phenotypic plants (Supplementary Table S4), implying that SNP A09, 900,112 co-segregated with the mutant phenotype. However, a recombinant was found at SNP A09, 1,723,490 of *BraA09g002790.3C*. The A:A genotype and the A:G genotype were detected in the mutant phenotypic plants. Thus, this SNP did not co-segregate with the mutant phenotype. These results confirm that *BraA09g001440.3C* harbors SNP A09, 900,112, and is the most likely candidate gene of the *nhm4-1* mutant. Gene annotation indicates that *BraA09g001440.3C* is a homologous gene of *Arabidopsis CPS1* (*At4g02780*) and encodes the CPS1 enzyme, which catalyzes the conversion of geranylgeranyl pyrophosphate (GGPP) to copalyl pyrophosphate (CPP) in GA biosynthesis. In this study, the candidate gene of the *nhm4-1* mutant is referred to as *BrCPS1*.

### Cloning and Sequence Analysis of *BrCPS1*

**Figure 4:**
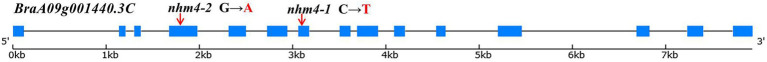
Gene structure of *BraA09g001440.3C*. The blue boxes represent exons and the black lines represent introns.

**Figure 5:**
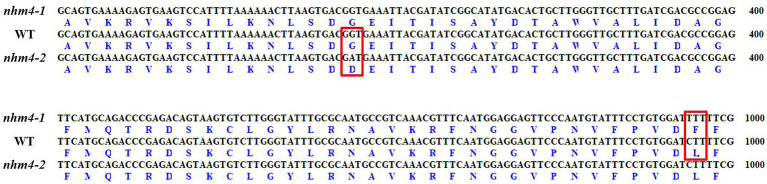
Alignment of coding and amino acid sequences of *BrCPS1*. The red frames show the sites where mutant *nhm4-1* and mutant *nhm4-2* occurred non-synonymous mutations.

Since MutMap and KASP analyses supported *BrCPS1* as the most likely candidate gene of *Brnhm4-1*, we cloned the coding sequence of *BrCPS1* from the wild-type “FT,” mutant *nhm4-1*, and mutant *nhm4-2*. Gene annotation showed that *BrCPS1* was 7,928 bp in length and consisted of 15 exons (Figure 4). Sequence alignment of the cDNAs and deduced amino acids between the wild-type and mutants is shown in Figure 5. Sequence comparison showed that a single base substitution occurred at position A09, 900,112 (C to T) in *nhm4-1*, resulting in an amino acid to change from leucine (L) to phenylalanine (F). However, the *BrCPS1* clone in mutant *nhm4-2* plants showed a single-nucleotide mutation in the fourth exon (A09, 898,783; G–A), resulting in an amino acid to change from glycine (G) to aspartic acid (D). This differs from the mutation site of the *nhm4-1* mutant, suggesting that *nhm4-1* and *nhm4-2* are allelic mutations of the same *BrCPS1* gene.

In this study, we also cloned the candidate gene *BraA09g002790.3C* (SNP A09, 1,723,490) in mutants *nhm4-1* and *nhm4-2*. There was a single base mutation (G–A) in *nhm4-1* at position A09, 1,723,490, which is consistent with the MutMap results. There was no change in the mutant *nhm4-2*. These results further confirm that *BrCPS1* is the gene responsible for the non-heading phenotype.

### Enzyme Activity Assays

To investigate whether the activity of CPS1 was changed in mutant *nhm4-1*, we measured the activity of CPS1 in wild-type and mutant *nhm4-1*. The activity of CPS1 was significantly decreased in the mutant *nhm4-1* compared with the wild-type (Supplementary Figure S2).

### Analysis of *BrCPS1* Expression Patterns by qRT-PCR

**Figure 6:**
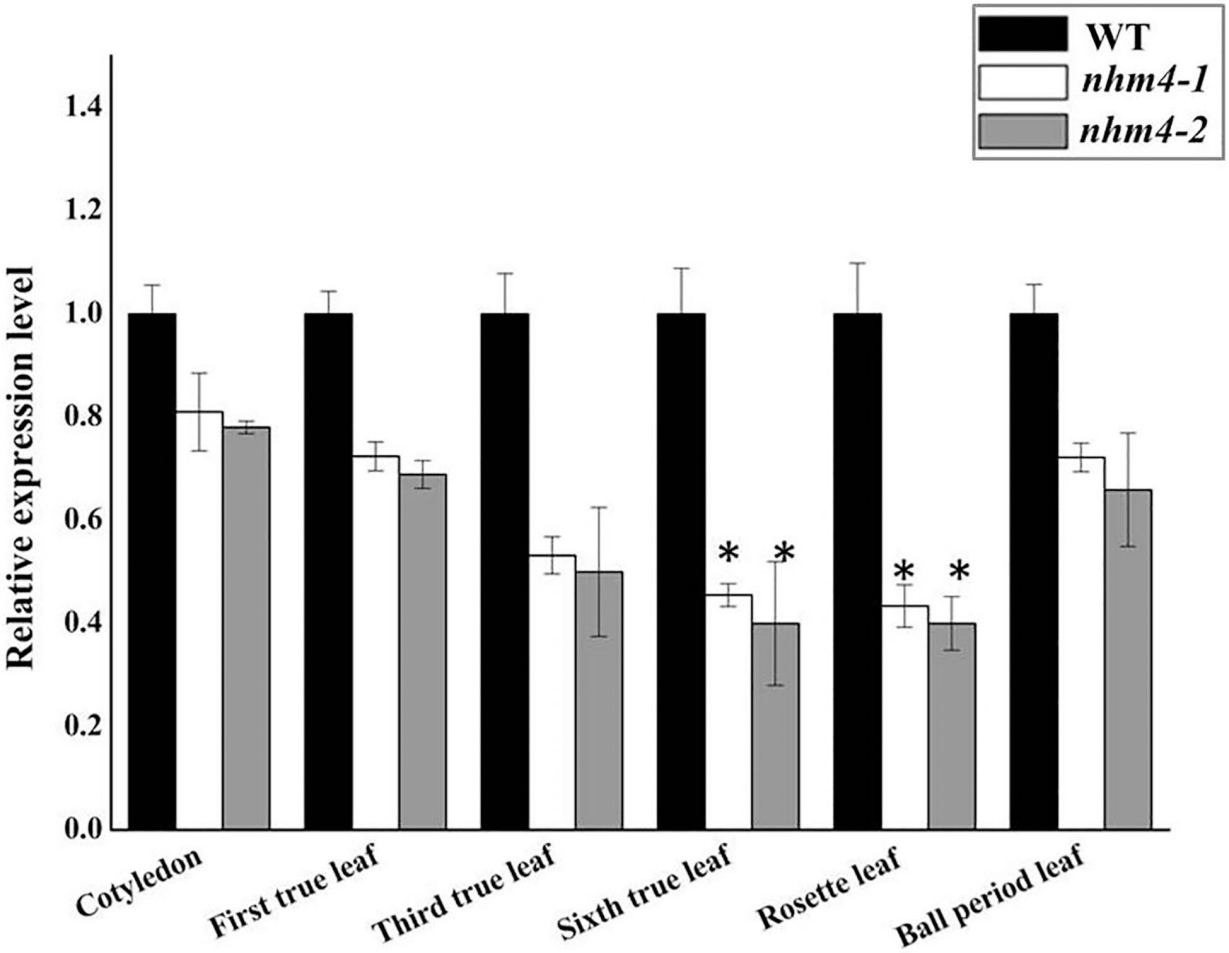
The expression levels of *BrCPS1*. Expression levels of genes in wild-type “FT” were used as reference for relative expression. ^*^ refers to significant differences in expression levels at *p* < 0.05 (Student’s *t*-test).

To investigate whether the mutation site affects gene expression, we used qRT-PCR to analyze the expression level of *BrCPS1* in the cotyledons, true leaves, rosette leaves, and heading leaves of wild-type “FT,” mutant *nhm4-1*, and mutant *nhm4-2* plants. The expression levels of *BrCPS1* were reduced at all stages of leaf development in both mutants, especially in the rosette leaves, which differ from wild-type “FT” results (Figure 6).

### Analysis of GA Content in Mutants *nhm4-1* and *nhm4-2*

**Figure 7:**
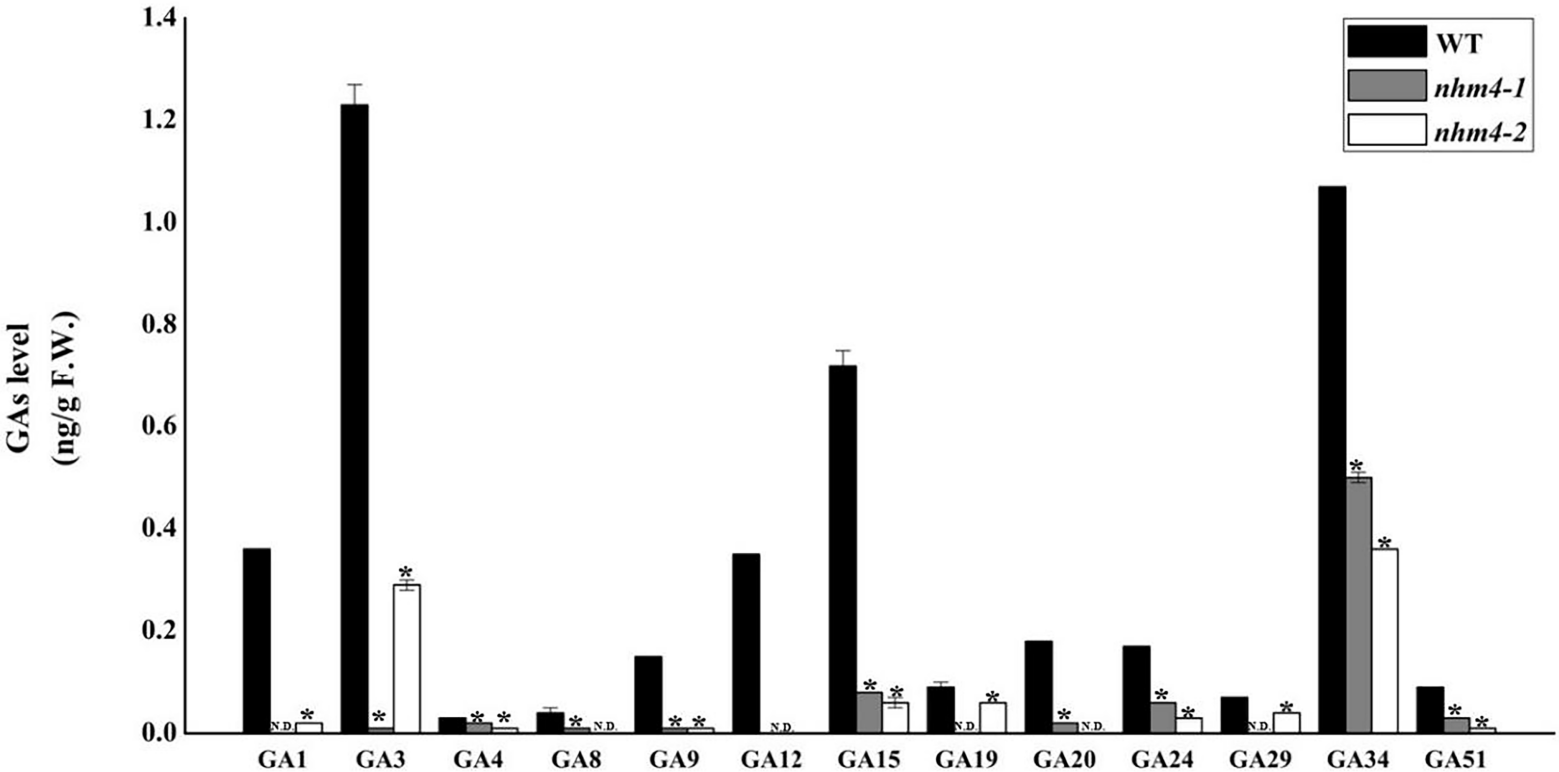
Determination of endogenous GA content in the leaves of wild-type “FT” and mutant plants. The content of GA in wild-type “FT” was used as control. ^*^ represents significant differences at *p* < 0.01 (Student’s *t*-test). N.D., not detectable.

We determined the GA content of 18 endogenous GAs (GA_1_, GA_3_, GA_4_, GA_5_, GA_6_, GA_7_, GA_8_, GA_9_, GA_12_, GA_15_, GA_19_, GA_20_, GA_24_, GA_29_, GA_34_, GA_44_, GA_51_, and GA_53_) in the leaves of wild-type and mutant plants. GA_5_, GA_6_, GA_7_, GA_44_, and GA_53_ were not detected in either the wild-type or mutant strains (Figure 7). GA_12_ was detected only in the wild-type and not in either of the mutants. The levels of GAs (GA_9_, GA_15_, GA_20_, and GA_24_) in the bioactive GA biosynthesis pathway were significantly decreased in the mutants than in the wild-type.

### Effects of Exogenous Application of GA_3_ on the Non-heading Phenotype

**Figure 8:**
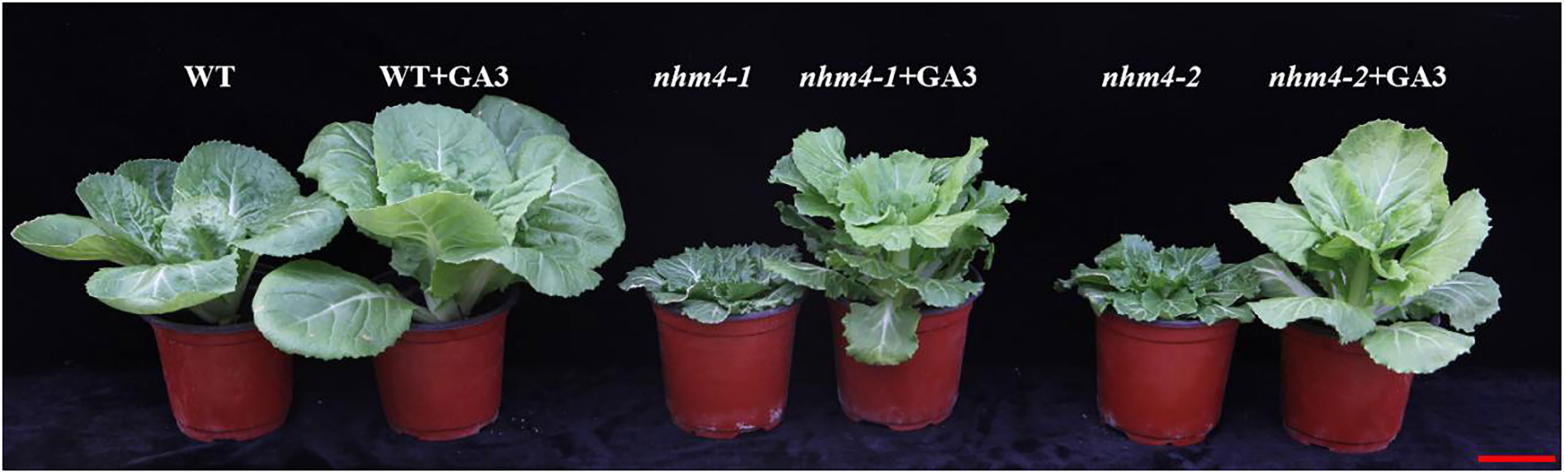
Phenotypic responses of *nhm4-1* and *nhm4-2* to exogenous GA_3_ application. Bar = 5 cm.

Based on the determination results of endogenous GA content, we investigated the responses of the mutants to exogenous GA_3_ application. After exogenous spraying of GA_3_, we observed that the leaves of the mutants had grown upward, similar to the wild-type leaves in the rosette stage, which demonstrates a tendency to form a leafy head (Figure 8). Consequently, leafy head formation is related to GA biosynthesis or the deactivation pathway.

## Discussion

Chinese cabbage is an important economic vegetable, and the formation of Chinese cabbage leafy heads significantly affects yield and quality. In this study, we identified a pair of allele non-heading mutants: *nhm4-1* and *nhm4-2*. Based on the MutMap, KASP, and map-based cloning results, we indicated that the mutation of *CPS1*, a key enzyme of GA biosynthesis, causes a non-heading phenotype. These results revealed that the formation of leafy heads is related to GA biosynthesis in Chinese cabbage.

Non-heading mutants are important resources for studying the mechanism of leafy head formation in Chinese cabbage. Selecting a suitable mutagenic material is an important prerequisite for the creation of mutants. Inbred lines are commonly used as mutagenic materials in *Brassica* crops (Barro et al., 2001; Ferrie et al., 2008; Liu et al., 2010). However, in this study, a heading Chinese cabbage double haploid (DH) line was employed as the mutagenic material to create non-heading mutants. The genetic background of the DH line was homozygous, which is beneficial for screening mutants. Moreover, the genetic background was highly consistent between the wild-type “FT” and the mutants, and genetic differences only occurred at the mutation sites, which is helpful for functional genomics investigation in plants.

The application of allelic mutants to identify gene functions has been proven to be effective in lettuce (Huo et al., 2016), sorghum (Jiao et al., 2018), maize (Zhao et al., 2018), and rice (Lee et al., 2019). Zhang et al. (2018) identified two round-leaf mutants, *rl-1* and *rl-2*, from an EMS mutagenic population in cucumber. The candidate for *rl-1* was identified as *CsPID*, which encodes a Ser/Thr protein kinase. The results of map-based cloning showed that the *rl-2* gene was also located on the same candidate gene *CsPID*, but the mutation site was different from that of *rl-1*. Allelism tests also confirmed that *rl-1* and *rl-2* were alleles. The use of allelic mutants *rl-1* and *rl-2* strongly proved that the *CsPID* gene is a candidate gene for controlling the formation of round leaves. As a reference, the plant materials in this study were two allelic mutants, and we used the same method to verify the function of the mutated gene.

Gibberellin is a diterpenoid plant hormone that has various regulatory effects on plant growth, such as germination, stem elongation, and flowering (Yamaguchi, 2008; Hedden and Thomas, 2012). *CPS* is the first gene that enters the GA biosynthesis pathway. Because of its key role in GA biosynthesis, many different *CPS* genes have been identified in species including *Arabidopsis thaliana* (Sun and Kamiya, 1994), *Zea mays* (Bensen et al., 1995), and *Oryza sativa* (Otomo et al., 2004; Prisic et al., 2004; Xu et al., 2004). However, in this study, based on the results of endogenous GA content determination, we speculated that the mutation of the *BrCPS1* gene caused the inability to produce and accumulate bioactive GAs (GA_1_, GA_3_, GA_4_, and GA_7_) in the leaves, which made the leaves appear to grow geotropically and thus could not form leafy heads. We conducted exogenous GA_3_ spraying experiments on the mutants, and found that after exogenous supplementation of GA_3_, the leaves of mutant grew upward similar to the wild-type at the rosette stage, which proved it has a tendency to form the leafy head and GA_3_ played a role in the process of leafy head formation, and the upward leaves are the necessary process of leafy head formation. Consistent with our previous research, the mutation of *KS*, an important enzyme, catalyzes the second step in the cyclization of GGPP into ent-kaurene in the GA synthesis pathway, also led to a non-heading phenotype of Chinese cabbage (Gao et al., 2020). Both mutated genes are key enzymes in the starting site of GA biosynthesis. Mutations in both genes resulted in decreased GA content in the mutants, and inhibited the leafy head formation, further demonstrating that the GA content in the leaves is an important factor affecting leafy head formation.

In general, combined with the results of previous studies, we believe that the important enzymes involved in the synthesis of GA have mutated, hindering the biosynthesis of GA and resulting in the inability to produce and accumulate biologically active GAs. The lack of GA in Chinese cabbage leaves prevents the formation of leafy heads. These results can contribute to further our understanding of the molecular mechanisms of Chinese cabbage leafy head formation.

## Data Availability Statement

The datasets presented in this study can be found in online repositories. The names of the repository/repositories and accession number(s) can be found at: https://www.ncbi.nlm.nih.gov/, SAMN26241760, https://www.ncbi.nlm.nih.gov/, SAMN26241761, https://www.ncbi.nlm.nih.gov/, SAMN26241762.

## Author Contributions

YG and GQ have equally contributed to this study. HF and YG designed the experiments. YG conducted the experiments, performed the data analysis, and wrote the manuscript. GQ, MZ, and ZL helped create the mutants. WF and SH assisted in the date analysis. HF revised the manuscript. All authors contributed to the article and approved the submitted version.

## Funding

The research was supported by the National Natural Science Foundation of China (Grant No. 31730082).

## Conflict of Interest

The authors declare that the research was conducted in the absence of any commercial or financial relationships that could be construed as a potential conflict of interest.

## Publisher’s Note

All claims expressed in this article are solely those of the authors and do not necessarily represent those of their affiliated organizations, or those of the publisher, the editors and the reviewers. Any product that may be evaluated in this article, or claim that may be made by its manufacturer, is not guaranteed or endorsed by the publisher.

## Footnotes

^1^ http://brassicadb.cn
