# Supplementary material for: BrCPS1 Function in Leafy Head Formation Was Verified by Two Allelic Mutations in Chinese Cabbage (Brassica rapa L. ssp. pekinensis)
Source: Front Plant Sci. 2022 Jul 12;13:889798. doi: 10.3389/fpls.2022.889798 (PMC9315314; doi:10.3389/fpls.2022.889798)
Supplement: Supplementary file 1 [file Data_Sheet_1.docx]

Supplementary Material

**Supplementary Table S1.** Primers used in KASP analyses

| Gene ID | Primer_AlleleX | Primer_AlleleY | Primer_Common |
| --- | --- | --- | --- |
| *BraA09g002790.3C* | GCGCTCCAGTTCAGCTTCG | CGCGCTCCAGTTCAGCTTCA | AATCGAAGCCCTAGGCGGATCC |
| *BraA09g001440.3C* | GTTCCCAATGTATTTCCTGTGGATC | AGTTCCCAATGTATTTCCTGTGGATT | TTGTAGGCGATCCACGATCCATATG |

**Supplementary Table S2.** Primers used for gene cloning

| Primer name | Primer Sequences (5′–3′) | Length of PCR products (bp) | Tm (°C) |
| --- | --- | --- | --- |
| *BraA09g002790.3C*-F | ATGAAGGAAGAGGAAGTAAACCGGT | 1005 | 60 |
| *BraA09g002790.3C*-R | TCAAACATCTTCATCATCCGACTCA |  |  |
| *BraA09g001440.3C*-F | ATGTCTCTACAATACTATGCTCTTAACTC | 2424 | 56 |
| *BraA09g001440.3C*-R | TTATAGAACTTTTTGAAACAAGATTTT |  |  |

**Supplementary Table S3.** Primers used for qRT-PCR

| Primer name | Primer Sequences (5′–3′) |
| --- | --- |
| *BrCPS1*-F | AATCAAACCAGGCGGTGACT |
| *BrCPS1-*R | CCCAATCTCGCCAGGTAAGT |

**Supplementary Table 4.** Results of KASP analyses

| F_2_ population | Phenotype | Genotype-SNP A09, 1,723,490 | Genotype-SNP A09, 900,112 | F_2_ population | Phenotype | Genotype-SNP A09, 1,723,490 | Genotype-SNP A09, 900,112 |
| --- | --- | --- | --- | --- | --- | --- | --- |
| 1 | mutant | A:A | T:T | 93 | wild-type | A:G | T:C |
| 2 | mutant | A:A | T:T | 94 | wild-type | A:G | T:C |
| 3 | mutant | A:A | T:T | 95 | wild-type | G:G | C:C |
| 4 | mutant | A:A | T:T | 96 | wild-type | G:G | C:C |
| 5 | mutant | A:A | T:T | 97 | wild-type | G:G | C:C |
| 6 | mutant | A:A | T:T | 98 | wild-type | A:G | T:C |
| 7 | mutant | A:A | T:T | 99 | wild-type | G:G | C:C |
| 8 | mutant | A:A | T:T | 100 | wild-type | A:G | T:C |
| 9 | mutant | A:A | T:T | 101 | wild-type | A:G | T:C |
| 10 | mutant | A:A | T:T | 102 | wild-type | A:G | T:C |
| 11 | mutant | A:G | T:T | 103 | wild-type | A:G | T:C |
| 12 | mutant | A:A | T:T | 104 | wild-type | A:G | T:C |
| 13 | mutant | A:A | T:T | 105 | wild-type | G:G | C:C |
| 14 | mutant | A:A | T:T | 106 | wild-type | A:G | T:C |
| 15 | mutant | A:A | T:T | 107 | wild-type | A:G | T:C |
| 16 | mutant | A:A | T:T | 108 | wild-type | A:G | T:C |
| 17 | mutant | A:A | T:T | 109 | wild-type | A:G | T:C |
| 18 | mutant | A:A | T:T | 110 | wild-type | G:G | C:C |
| 19 | mutant | A:A | T:T | 111 | wild-type | A:G | T:C |
| 20 | mutant | A:A | T:T | 112 | wild-type | G:G | C:C |
| 21 | mutant | A:A | T:T | 113 | wild-type | G:G | C:C |
| 22 | mutant | A:A | T:T | 114 | wild-type | A:G | T:C |
| 23 | mutant | A:A | T:T | 115 | wild-type | A:G | T:C |
| 24 | mutant | A:A | T:T | 116 | wild-type | A:G | T:C |
| 25 | mutant | A:A | T:T | 117 | wild-type | A:G | T:C |
| 26 | mutant | A:A | T:T | 118 | wild-type | A:G | T:C |
| 27 | mutant | A:A | T:T | 119 | wild-type | G:G | C:C |
| 28 | mutant | A:A | T:T | 120 | wild-type | G:G | C:C |
| 29 | mutant | A:A | T:T | 121 | wild-type | G:G | C:C |
| 30 | mutant | A:A | T:T | 122 | wild-type | A:G | T:C |
| 31 | mutant | A:A | T:T | 123 | wild-type | G:G | C:C |
| 32 | mutant | A:A | T:T | 124 | wild-type | G:G | C:C |
| 33 | mutant | A:A | T:T | 125 | wild-type | G:G | C:C |
| 34 | mutant | A:A | T:T | 126 | wild-type | A:G | T:C |
| 35 | mutant | A:A | T:T | 127 | wild-type | A:G | T:C |
| 36 | mutant | A:A | T:T | 128 | wild-type | G:G | C:C |
| 37 | mutant | A:A | T:T | 129 | wild-type | A:G | T:C |
| 38 | mutant | A:A | T:T | 130 | wild-type | A:G | T:C |
| 39 | mutant | A:A | T:T | 131 | wild-type | A:G | T:C |
| 40 | mutant | A:A | T:T | 132 | wild-type | G:G | C:C |
| 41 | mutant | A:A | T:T | 133 | wild-type | A:G | T:C |
| 42 | mutant | A:A | T:T | 134 | wild-type | A:G | T:C |
| 43 | mutant | A:A | T:T | 135 | wild-type | A:G | T:C |
| 44 | mutant | A:A | T:T | 136 | wild-type | A:G | T:C |
| 45 | mutant | A:A | T:T | 137 | wild-type | A:G | T:C |
| 46 | mutant | A:A | T:T | 138 | wild-type | G:G | C:C |
| 47 | mutant | A:A | T:T | 139 | wild-type | A:G | T:C |
| 48 | mutant | A:A | T:T | 140 | wild-type | A:G | T:C |
| 49 | wild-type | A:G | T:C | 141 | wild-type | A:G | T:C |
| 50 | wild-type | G:G | C:C | 142 | wild-type | A:G | T:C |
| 51 | wild-type | A:G | T:C | 143 | wild-type | A:G | T:C |
| 52 | wild-type | A:G | T:C | 144 | wild-type | G:G | C:C |
| 53 | wild-type | G:G | C:C | 145 | wild-type | A:G | T:C |
| 54 | wild-type | G:G | C:C | 146 | wild-type | A:G | T:C |
| 55 | wild-type | A:G | T:C | 147 | wild-type | A:G | T:C |
| 56 | wild-type | G:G | C:C | 148 | wild-type | G:G | C:C |
| 57 | wild-type | A:G | T:C | 149 | wild-type | A:G | T:C |
| 58 | wild-type | A:G | T:C | 150 | wild-type | A:G | T:C |
| 59 | wild-type | G:G | C:C | 151 | wild-type | G:G | C:C |
| 60 | wild-type | A:G | T:C | 152 | wild-type | A:G | T:C |
| 61 | wild-type | A:G | T:C | 153 | wild-type | G:G | C:C |
| 62 | wild-type | A:G | T:C | 154 | wild-type | G:G | C:C |
| 63 | wild-type | A:G | T:C | 155 | wild-type | G:G | C:C |
| 64 | wild-type | A:G | T:C | 156 | wild-type | A:G | T:C |
| 65 | wild-type | A:G | T:C | 157 | wild-type | G:G | C:C |
| 66 | wild-type | G:G | C:C | 158 | wild-type | G:G | C:C |
| 67 | wild-type | A:G | T:C | 159 | wild-type | G:G | C:C |
| 68 | wild-type | A:G | T:C | 160 | wild-type | A:G | T:C |
| 69 | wild-type | A:G | T:C | 161 | wild-type | G:G | C:C |
| 70 | wild-type | A:G | T:C | 162 | wild-type | G:G | C:C |
| 71 | wild-type | A:G | T:C | 163 | wild-type | G:G | C:C |
| 72 | wild-type | A:G | T:C | 164 | wild-type | A:G | T:C |
| 73 | wild-type | A:G | T:C | 165 | wild-type | A:G | T:C |
| 74 | wild-type | A:G | T:C | 166 | wild-type | A:G | T:C |
| 75 | wild-type | A:G | T:C | 167 | wild-type | G:G | C:C |
| 76 | wild-type | G:G | C:C | 168 | wild-type | A:G | T:C |
| 77 | wild-type | A:G | T:C | 169 | wild-type | A:G | T:C |
| 78 | wild-type | A:G | T:C | 170 | wild-type | A:G | T:C |
| 79 | wild-type | A:G | T:C | 171 | wild-type | A:G | T:C |
| 80 | wild-type | A:G | T:C | 172 | wild-type | A:G | T:C |
| 81 | wild-type | A:G | T:C | 173 | wild-type | G:G | C:C |
| 82 | wild-type | G:G | C:C | 174 | wild-type | A:G | T:C |
| 83 | wild-type | A:G | T:C | 175 | wild-type | A:G | T:C |
| 84 | wild-type | A:G | T:C | 176 | wild-type | A:G | T:C |
| 85 | wild-type | A:G | T:C | 177 | wild-type | A:G | T:C |
| 86 | wild-type | A:G | T:C | 178 | wild-type | A:G | T:C |
| 87 | wild-type | A:G | T:C | 179 | wild-type | A:G | T:C |
| 88 | wild-type | A:G | T:C | 180 | wild-type | A:G | T:C |
| 89 | wild-type | A:G | T:C | 181 | wild-type | A:G | T:C |
| 90 | wild-type | A:G | T:C | 182 | wild-type | A:G | T:C |
| 91 | wild-type | A:G | T:C | 183 | wild-type | A:G | T:C |
| 92 | wild-type | G:G | C:C | 184 | wild-type | G:G | C:C |


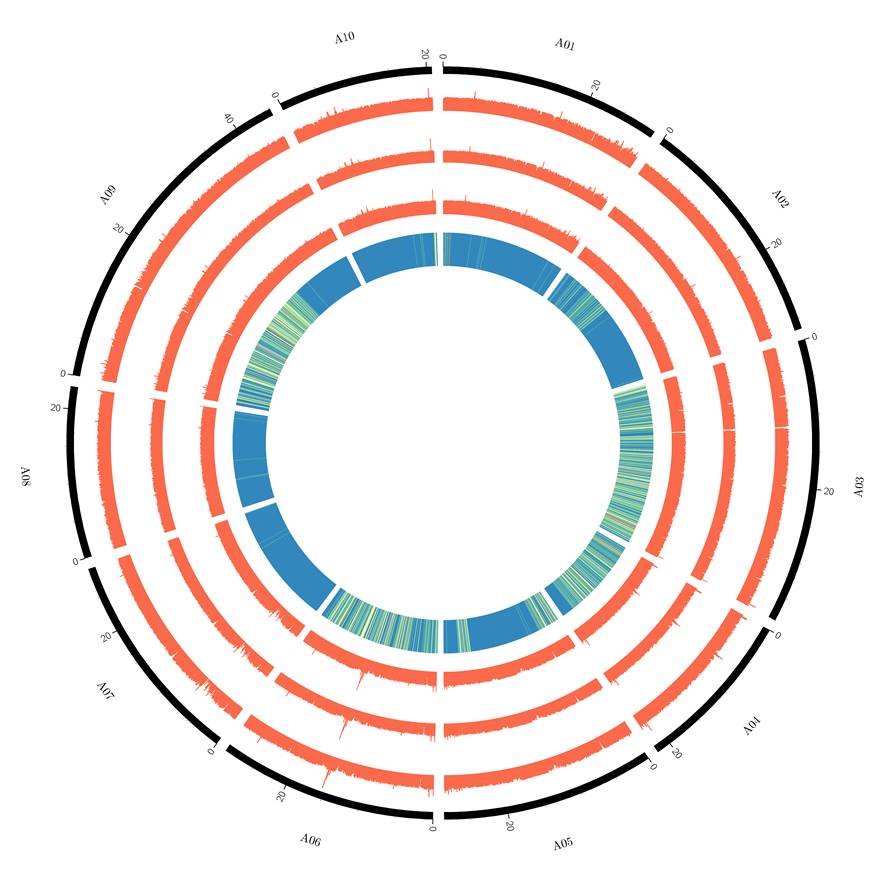


**Supplementary Figure S1. SNP density distribution.**

**
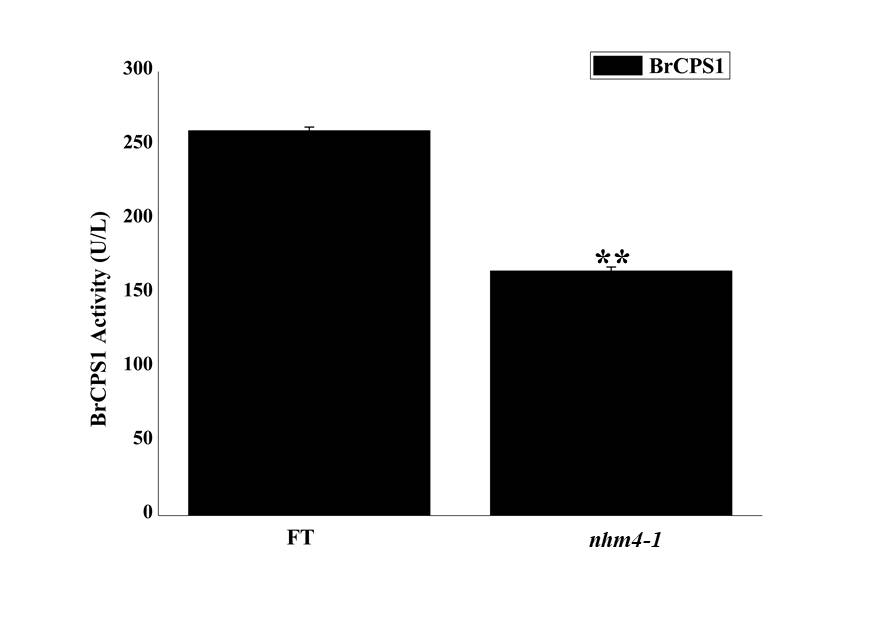
**

**Supplementary Figure S2. CPS1 activity assay in the wild-type ‘FT’ and *nhm4-1*.**
